# Single and Multiple Dose PK–PD Characterization for Carisoprodol. Part I: Pharmacokinetics, Metabolites, and 2C19 Phenotype Influence. Double-Blind, Placebo-Controlled Clinical Trial in Healthy Volunteers

**DOI:** 10.3390/jcm11030858

**Published:** 2022-02-06

**Authors:** Aitana Calvo, Saioa Alonso, Esther Prieto, Ana Ascaso-del-Rio, Jordi Ortuño, Nieves Fernandez, Antonio Portolés

**Affiliations:** 1 Clinical Pharmacology Department, Hospital Clínico San Carlos, IdISSC, 28040 Madrid, Spain; aitanacalvo@hotmail.com (A.C.); saioamuri@yahoo.es (S.A.); draestherprieto@gmail.com (E.P.); ana.ascaso@salud.madrid.org (A.A.-d.-R.); 2 Anapharm Bioanalytics, 08038 Barcelona, Spain; jortuno@anapharmbioanalytics.com; 3 Belmac Laboratories, 28700 Madrid, Spain; nfernandez@faes.es; 4 Pharmacology & Toxicology Department, Facultad de Medicina, Universidad Complutense de Madrid, 28040 Madrid, Spain

**Keywords:** carisoprodol, meprobamate, pharmacokinetics, phenotype, 2C19, clinical trial, drug abuse liability, central muscle relaxants

## Abstract

Carisoprodol was authorised in 1959 without a full pharmacokinetic–pharmacodynamic (PK–PD) characterisation. We designed a crossover, double-blind, placebo-controlled, randomized clinical trial to characterize the PKs of carisoprodol and its main active metabolite, meprobamate, after single (350 mg), multiple (350 mg/8 h, 14 days), and double (700 mg) doses of carisoprodol. Thirteen healthy volunteers were enrolled. After a single (350 mg) dose, the main carisoprodol parameters were (mean ± SD) Cmax: 2580 ± 1214 ng/mL, AUC_0–∞_: 8072 ± 6303 h·ng/mL, and half-life (T_1/2_): 2 ± 0.8 h. For meprobamate, the parameters were Cmax: 2181 ± 605 ng/mL and 34,529 ± 7747 h·ng/mL y 9 ± 1.9 h. Different profiles were found for extensive and poor 2C19 metabolizers. After 14 days of treatment (350 mg/8 h) the results for carisoprodol were (mean ± SD) Cmax: 2504 ± 730 ng/mL, AUC_0–∞_: 7451 ± 3615 h·ng/mL, and T_1/2_: 2 ± 0.7 h. For meprobamate (a steady state was reached), the parameters were Cmax: 5758 ± 1255 ng/mL and 79,699 ± 17,978 h·ng/mL y 8.7 ± 1.4 h. The study allowed for the full characterization of the pharmacokinetic profile of carisoprodol and meprobamate. Accumulation of meprobamate but not of carisoprodol was evident after 14 days of treatment.

## 1. Introduction

Pharmacokinetic (PK) factors play an important role in predicting the abuse liability and dependence of drugs. The PK characteristics that seem to contribute to dependence include long half-life, low free drug clearance, and sufficient concentrations in the body to allow tolerance to appear [1]. Rapid absorption and rapid delivery of psychoactive drugs to the central nervous system (CNS) appear to contribute to abuse and self-administration [2]. 

Therefore, the PK properties of potentially psychoactive drugs need to be clearly defined before drugs are placed on the market. However, the term “pharmacokinetic” was first introduced by Dost in 1953, and the first review on the matter was published in 1961 by Nelson [3]. Therefore, even though there was a transformation in the pharmaceutical industry due to new regulatory and technological conditions during the 1950–1960 decade [4], psychoactive drugs with inherent abuse potential due to their modes of action were authorised at the time without being fully characterised from a PK or pharmacodynamic (PD) perspective.

Carisoprodol is a centrally acting muscle relaxant that was authorised for the first time in 1959, both in the US and European markets, for the short-term treatment of acute, painful musculoskeletal conditions. Carisoprodol mainly treats lower back pain, often as adjunct to NSAIDs or acetaminophen. Although the mode of action of Carisoprodol is not fully characterized, in humans its effect appears to be mediated by sedation. Both meprobamate, carisoprodol’s active metabolite, and carisoprodol itself seem to have an impairing effect.

Recently, the FDA has included the control of rapid-acting opioids as a means of addressing the current crisis of abuse and misuse of opioids [5]. Europe, although not facing a crisis of the same dimensions as the US [6,7], has evaluated risks [8] and taken fragmentated actions especially for immediate release substances [9]. Obviously, the problems regarding drug abuse and misuse do not exclusively relate to opioids, as the illicit use and abuse of carisoprodol has increased in the last 20 years. According to the 2012 National Survey on Drug Use and Health data, 3.69 million people used carisoprodol for non-medical reasons, including diversion and trafficking, which are indicative of drug abuse [10]. In the European region, a safety revision led in 2007 to the recommendation of the suspension of carisoprodol throughout Europe [11], and in the US a similar process led in 2011 to the placement of carisoprodol into Schedule IV of the Controlled Substances Act [12]. The latter led to an initial moderate reduction in the dispensing of carisoprodol, especially among younger age groups and patients with injuries [13]. Its decline in use has been steady since 2014 [14]. Nonetheless, carisoprodol was among the top 350 drugs prescribed in the US in 2019 [14]. 

We designed a clinical trial to characterize the pharmacokinetics of carisoprodol and its main active metabolite, meprobamate, after single and multiple doses of carisoprodol, as PK studies had only been performed after single doses. Thus, an attempt to identify the PK characteristics that might contribute to carisoprodol’s dependence and abuse was performed. In addition, the study explored the pharmacodynamics of carisoprodol, and its results are presented in Part II of this publication.

## 2. Materials and Methods

Healthy subjects were selected with the following inclusion criteria: male or female subjects; age between 18 and 40 years; women of childbearing potential must have a negative pregnancy test prior to enrolment and must agree to use an effective contraceptive method during the study (other than oral contraceptives); body weight from 50–100 kg and body mass index from 18–27 (Quetelet index = weight in kg/height in m2); absence of any clinically significant deviation from normal in the physical or electrocardiographic examinations or medically significant values outside the normal range in clinical laboratory tests; and uninfluenced willingness to participate in the study documented by means of a written, signed, and dated informed consent form. Subjects should not meet any of the following exclusion criteria: smoking within six weeks prior to study inclusion; pregnancy or lactation; history of drug abuse or alcoholism and/or use of any commonly abused drugs within a month prior to study entry; high consumption of stimulating drinks (equivalent to 400 mg of caffeine per day, a cup of coffee approximately containing 100 mg of caffeine); use of any medication that could interfere with the study aim; participation in a clinical trial within 3 months prior to inclusion or participation in 4 clinical trials during the last year; history of significant medical conditions or major surgeries in the past 3 months; inability to cooperate with the investigators; history of drug allergies; diseases which can alter drug’s absorption, distribution, metabolism, and/or excretion (malabsorption, oedemas, and liver or renal impairment); HIV or hepatitis B or C infection; blood loss or blood donation (over 200 mL within 3 months); blood and blood products transfusion in the past 6 months; strenuous physical exercise within 72 h prior to admission; alcohol consumption within 48 h prior to admission. A sample size of at least 12 healthy subjects (foreseen 14) was considered enough for assessing the endpoints.

A double-blind, placebo-controlled, randomized, crossover, 2-period design was used to characterize the PK profile of carisoprodol and meprobamate after a single and multiple doses (14 days) of carisoprodol and on an exploratory basis for double doses (700 mg) on D7. The study was controlled with placebo in order to limit the occurrence of unconscious bias in the performance, conduct, and interpretation of pharmacodynamic outcomes, a secondary objective of the study.

**Figure 1:**
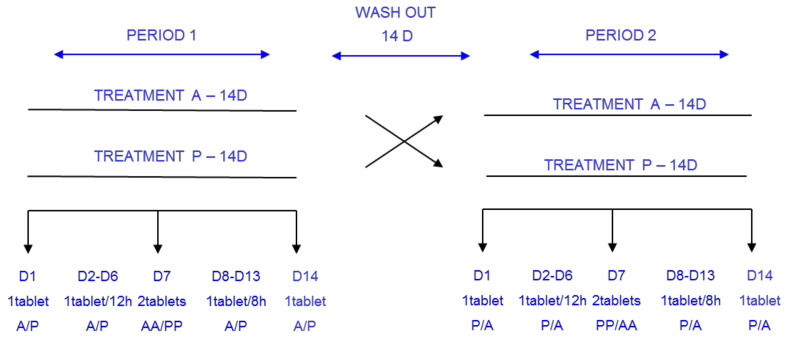
Study procedures schema (A: Active treatment (carisoprodol); P: Placebo; AA: double active treatment dose (carisoprodol 700 mg); PP: double placebo dose).

Carisoprodol (350 mg tablets) or placebo was to be taken orally on Day 1, then at increasing doses (every 12 h for 6 days and every 8 h for a further 6 days). A double dose of carisoprodol or placebo was administered on Day 7. The placebo tablet was mainly composed of lactose (and excipients). After a minimum washout period of 14 days, the volunteers were crossed over to the alternate arm (sequences: Active–Placebo, Placebo–Active) (see Figure 1). Assignment of subjects to the treatment sequence was blindly designed by a computer-generated randomization table, balanced by blocks. On Day 7 of each period a dose of 700 mg of Carisoprodol or placebo was administered. Due to the limited sample size, and because treatment received from Day 1 to Day 6 would influence PK (and PD) parameters for Day 7, another randomization sequence was performed for Day 7, and thus 4 different possibilities of exposure to active treatment resulted: Arm A-AA (Days 1 to 6 of active treatment (A), which corresponds to a single dose of 350 mg of Carisoprodol every 6–8 h, accordingly, then on, Day 7, a double dose of active treatment (AA), which corresponds to 700 mg of a single dose of Carisoprodol); Arm A-PP (Days 1–6, single dose of active treatment (A); Day 7 double dose of placebo, PP); Arm P-AA (Days 1–6, single dose of placebo every 6–8 h (P); Day 7 double dose of active treatment AA); Arm P-PP (Days 1–6 single dose of placebo (P), Day 7 double dose of placebo). 

The increase in the dosage was progressive in order to facilitate the tolerability of the treatment and to avoid dropouts.

Blood samples were drawn on D1 and D14 at the following time points: baseline, +0.5, +1, +1.5, +2, +3, +4, +5, +6, +7, +8, +10, +12, and +24. Additionally drawn on D7: baseline, +0.5, +1, +1.5, +2, +3, +4, and +5; baseline samples were also drawn on Days 12 and 13. The plasma concentration values were entered into a program for kinetic analysis (WinNonlin, release 4.1) for individual estimation of the drug parameters. PK samples on D14 were obtained in order to determine PKs at steady state at the maximum length of treatment recommended according to carisoprodol’s specification (11) and to explore PD parameters at this time point. 

PK parameters for carisoprodol and meprobamate included AUClast (area under the time/plasma concentration curve, calculated by using the linear trapezoidal method from the base to the last measurable concentration); AUC_0–∞_ (AUClast corrected for an additional area calculated from the last measurable concentration); AUC_0–8_ (AUC from baseline to +8 h); AUC_0–12_ (AUC from the baseline to +12 h); Cmax (maximum plasma concentration); Tmax (time to reach the Cmax); and T1/2: plasma half-life time, after single (Day 1) and multiple doses (Day 14) as well as for double-doses (Day 7). 

Concentrations of carisoprodol and meprobamate in plasma samples were measured by reversed phase high performance liquid chromatography coupled to a tandem mass spectrometry detector (LC/MS/MS) (see Appendix A).

The phenotypic condition of extensive (EM) or poor metabolizer (PM) was obtained upon the analysis of the CYP2C19 genotype’s polymorphism.

PK parameters were examined using a non-compartmental method. Descriptive statistics were used to describe the parameters. PD parameters were explored, and the study was executed as a single PK–PD study. Given the complexity and extension of all the procedures, it has been divided in 2 parts (I: PK; II: PD).

The study was performed according to the rules for good clinical practice and was authorized by the Ethics Committee of the Hospital Clínico San Carlos (IRB registry: 06/230) and by the Spanish agency on Medicines and Sanitary Products (Clinical Trial Registry, EudraCT: 2006-004254-24). The ethics principles of the Declaration of Helsinki and its further revisions, together with the principles of Good Clinical Practice, were observed, with written informed consent given by each subject prior to any study procedure.

## 3. Results

**Figure 2:**
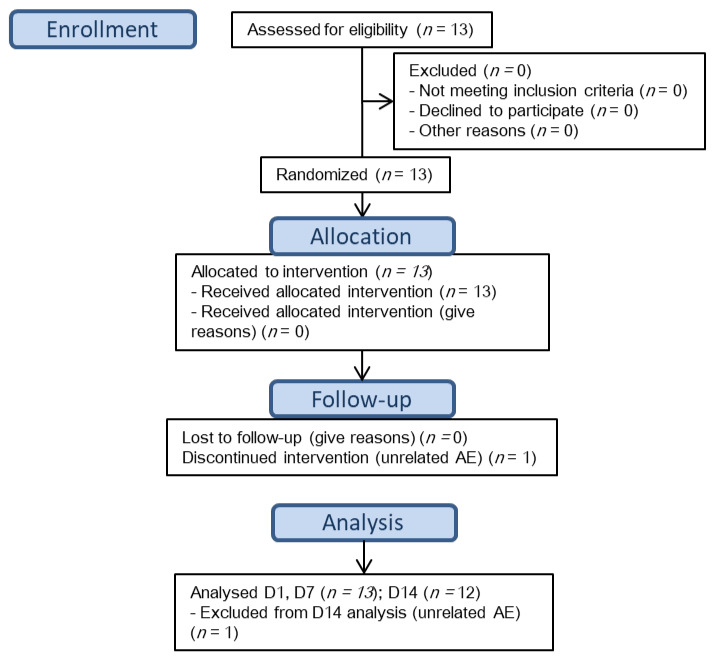
Patient Flow Chart.

Thirteen healthy subjects (8 male, 5 female) were recruited, twelve finished the study of PK procedures (1 dropped out before D14) (Figure 2, patient flow chart as per CONSORT 2010). The demographic characteristics are summarized in Table 1.

**Figure 3:**
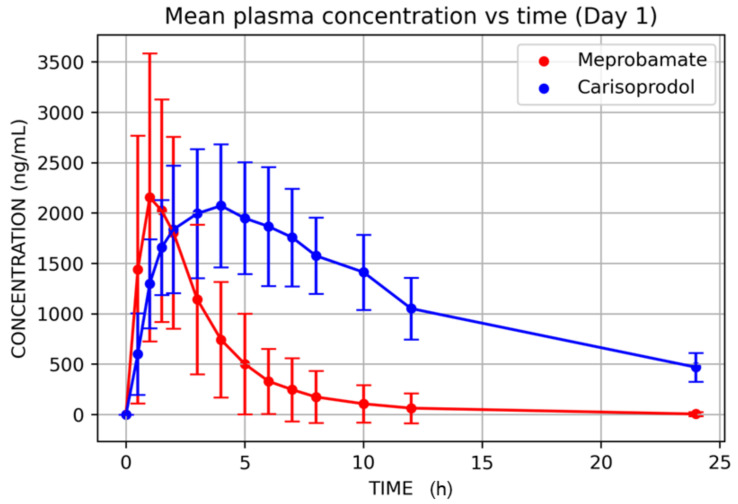
Carisoprodol and meprobamate mean plasma concentrations after single carisoprodol 350 mg doses.

**Figure 4:**
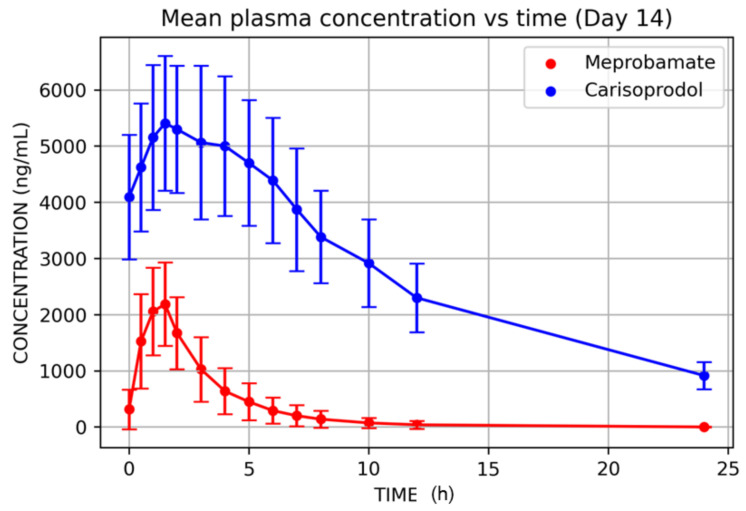
Mean plasma concentrations of carisoprodol and meprobamate after multiple carisoprodol dosing (350 mg/8 h, 14 days).

Mean plasma carisoprodol and meprobamate concentrations versus time after single (D1) and multiple doses (D14) are displayed in Figure 3 and Figure 4, and their PK parameters shown in Table 2 and Table 3.

The pharmacokinetics of carisoprodol and meprobamate were adequately characterized after single and multiple doses of carisoprodol. The Cmax (mean ± SD) after a single dose was 2580 ± 1214 ng/mL; the AUC_0–∞_ was 8072 ± 6303 h·ng/mL; and the T_1/2_ was 2 ± 0.8 h for carisoprodol. For meprobamate, these values were 2181 ± 605 ng/mL; 34,529 ± 7747 h·ng/mL and 9 ± 1.8 h, respectively. 

Concerning the multiple-dose treatment, baseline carisoprodol and meprobamate concentrations were similar on D12, D13, and D14, with no statistically significant differences between D12, or D13, and D14. Thus, it is acceptable to deem a steady state reached by D14.

Results for carisoprodol were very similar for D14 vs. D1, showing the following figures: Cmax was 2504 ± 730 ng/mL; AUC_0–∞_: 7451 ± 3615 h·ng/mL; and T_1/2_: 2 ± 0.7 h. Due to its longer half-life, meprobamate accumulated during the multiple-dose period, up to a steady state, and Cmax and AUC values on D14 were significantly higher than on D1 although no difference was found for T_1/2_ (*p*: 0.85). The figures were: Cmax: 5758 ± 1255 ng/mL; 79,699 ± 17,978 h·ng/mL y 8.7 ± 1.4 h.

**Figure 5:**
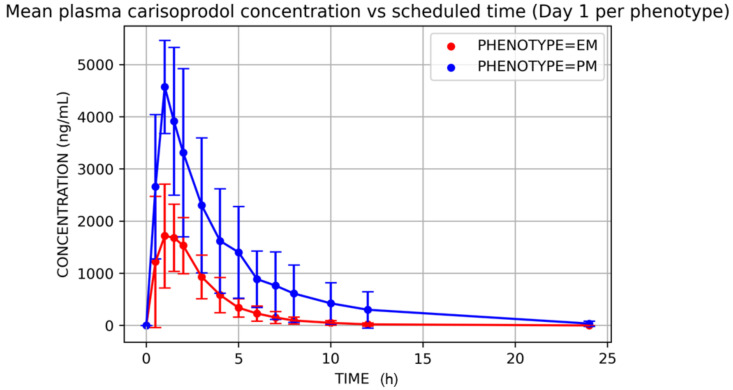
Mean plasma carisoprodol concentration vs. time, D1, by phenotype. EM = extensive metabolizer; PM = poor metabolizer.

**Figure 6:**
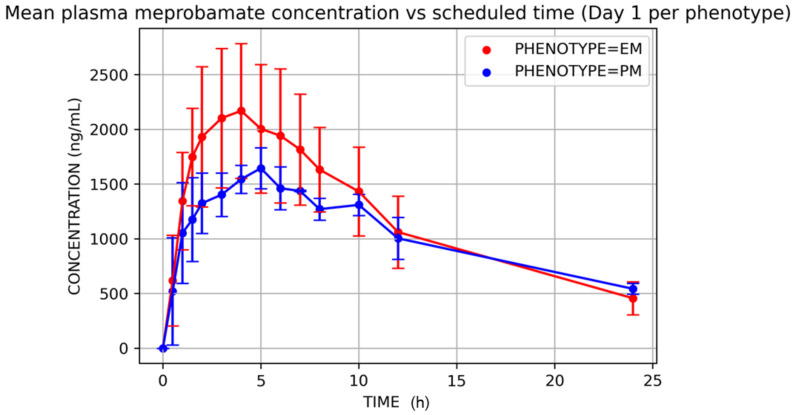
Mean plasma meprobamate concentration vs. time, D1, by phenotype. EM = extensive metabolizer; PM = poor metabolizer.

Poor metabolizers showed lower carisoprodol clearance (mean ± SD, mL/h; *t*-test *p*-value) (21,585.61 ± 12,130.85 vs. 71,373.66 ± 39,441.61; *p* = 0.01) on D1, which resulted in higher carisoprodol concentrations (in comparison to EMs). Consequently, meprobamate concentrations were higher for EMs (Figure 5 and Figure 6). AUC_0–∞_ on D1 were (mean ± SD, ng·h/mL) for carisoprodol 6038 ± 2530 vs. 19,255 ± 10,821 for extensive and poor metabolizers, respectively, and for meprobamate they were 34,694 ± 8446 vs. 33,623 ± 2202 for extensive and poor metabolizers, respectively.

Pharmacokinetic results for the double dose on Day 7 are displayed in the Supplementary Materials (Figures S1 and S2, Tables S1 and S2), as four groups with a limited sample size each due to previous placebo or drug exposures had to be reported, and thus results can only be considered exploratory; they are also displayed there in order to allow more exposition for pharmacodynamics evaluation.

## 4. Discussion

Safety problems recognized after published case reports of abuse of and dependence on carisoprodol [15,16,17,18,19,20,21,22,23], together with the publication of data by the Norwegian Prescription Agency which detected the use of carisoprodol at doses higher than recommended, commonly in association with benzodiazepines and opioids [24], and data from the public system of the US which monitors the visits to the E.R. departments related to drugs, led to the suspension of the authorisation of its commercialization in the European region [11]. In the US, carisoprodol was placed into Schedule IV of the Controlled Substances Act [10].

PK factors play an important role in predicting the abuse liability and dependence of drugs. However, carisoprodol was authorised in 1959 without having been fully characterised from a PK or PD perspective according to current standards. In fact, PK data after multiple doses of carisoprodol have not yet been reported.

The PK characteristics that seem to contribute to dependence include long half-life, low free drug clearance and sufficient concentrations in the body to allow tolerance to appear [1]. Rapid absorption and rapid delivery of the drug to the central nervous system (CNS) appear to contribute to abuse and self-administration [2]. The current opioid crisis, in which immediate-release opioids play a major role, increases the interest in characterising the PK characteristics of a psychoactive drug with a recognised abuse potential. 

Our study allowed a full characterization of the PK profile of carisoprodol and its metabolite, meprobamate, after single and multiple doses of 350 mg/8 h (14 days of treatment). It also showed the effect of poor 2C19 metabolizing and explored all parameters after double doses, which are the maximum recommended dose (700 mg). PK parameters after a single dose of 350 mg were similar to those described previously [25,26,27,28]. The PK of carisoprodol after multiple doses had not been previously reported. In our study, there was no evidence of relevant accumulation of carisoprodol during 14 days of treatment at maximum authorized doses. However, baseline meprobamate concentrations were detected on Day 14, together with higher Cmax and AUC values on Day 14. T_1/2_ was similar on Days 1 and 14, indicating that linear pharmacokinetics are maintained at least for this dosing schedule. Given the repeated dosing and the elimination half-life of meprobamate, its concentrations increased up to a steady state (SS), presumably reached on the second or third day of treatment. The comparison of the baseline concentration in the previous days to D14 confirms SS was reached.

The washout period was long enough that baseline concentrations were undetectable on D1 of the second period. 

Two out of thirteen subjects (15%) were classified phenotypically as slow metabolizers for substrates of CYP2C19. This percentage is higher than that previously reported for the Spanish population (2–5%) [29,30]; this difference could be due to the limited sample size. The Cmax and AUC for carisoprodol were clearly higher among poor metabolizers, while meprobamate’s Cmax and AUC were higher for extensive metabolizers. These findings suggest that CYP2C19 is the main isoenzyme for carisoprodol metabolism [24,26,31], though the clinical relevance of this finding should be analysed because no differences have been found in genetic mutations of CYP2C19 in fatal carisoprodol intoxications [32].

Concerning the highest single doses, 700 mg of carisoprodol or placebo were randomly administered on D7 in an attempt not only to describe PKs after a double dose, but also in order to further explore PD effects (described in part II). Incomplete randomization was done, as, with our insufficient sample size, it was not possible to explore all sequences.

Pharmacodynamics were also explored (and will be published elsewhere; Part II, presently under review), allowing for the most complete evaluation of PK–PD characterization. 

## 5. Conclusions

The study allowed for full characterization of the pharmacokinetic profile of carisoprodol and its main metabolite, meprobamate. Accumulation of meprobamate but not of carisoprodol was evident after multiple doses; both compounds show linear pharmacokinetics. Our study also confirmed the primary role of CYP2C19 in the pharmacokinetics of meprobamate.

## Figures and Tables

**Table 1:** Demographic characteristics of subjects.

	Age	Weight (kg)	Height (cm)	Quetelet Index (kg/m^2^)
Mean	23.076	69.16	173	22.89
SD	1.07	12.85	8.13	2.57

**Table 2:** Pharmacokinetic parameters of carisoprodol after single (350 mg) or multiple doses (up to 350 mg/8 h, 14 days).

**Pharmacokinetic Parameters of Carisoprodol after Single (350 mg) or Multiple Doses (up to 350 mg/8 h, 14 days)**								
**Day 1**								
**Variable**	***N***	**Mean**	**SD**	**Min**	**Median**	**Max**	**CV%**	**Geometric** **Mean**
Tmax (h)	13	1.19	0.69	0.50	1.00	3.00	58.15	1.04
Cmax (ng/mL)	13	2579.82	1214.05	935.14	2294.50	5204.37	47.06	2316.47
AUC_0–8_ (ng·h/mL)	13	7219.99	4550.97	2283.54	6070.36	19,807.26	63.03	6165.38
AUC_0–12_ (ng·h/mL)	13	7671.52	5276.52	2334.17	6404.65	22,762.02	68.78	6427.87
AUClast (ng·h/mL)	13	7955.50	6184.89	2322.03	6404.65	26,473.74	77.74	6498.54
AUC_0–∞_ (ng·h/mL)	13	8071.70	6303.04	2348.37	6448.63	26,906.93	78.09	6578.31
T_1/2_ (h)	13	1.98	0.84	1.06	1.67	4.19	42.55	1.85
**Day 14**								
**Variable**	***N***	**Mean**	**SD**	**Min**	**Median**	**Max**	**CV%**	**Geometric** **Mean**
Tmax (h)	12	1.08	0.42	0.50	1.00	1.50	38.53	1.00
Cmax (ng/mL)	12	2503.58	730.08	1408.42	2533.82	3556.65	29.16	2397.05
AUC_0–8_ (ng·h/mL)	12	6905.29	2793.14	3815.50	6471.21	14,151.96	40.45	6477.15
AUC_0–12_ (ng·h/mL)	12	7233.72	3174.64	3917.30	6776.70	15,668.25	43.89	6727.29
AUClast (ng·h/mL)	12	7362.86	3582.20	3917.30	6776.70	17,299.65	48.65	6774.03
AUC_0–∞_ (ng·h/mL)	12	7451.07	3614.80	3953.15	6918.11	17,425.04	48.51	6854.52
T_1/2_ (h)	12	1.98	0.73	1.27	1.84	3.62	36.75	1.87

**Table 3:** Meprobamate pharmacokinetic parameters after single (350 mg) or multiple carisoprodol doses (up to 350 mg/8 h. 14 days).

**Pharmacokinetic Parameters of Meprobamate after Single (350 mg) or Multiple Doses of Carisoprodol (up to 350 mg/8 h, 14 Days)**								
**Day 1**								
**Variable**	***N***	**Mean**	**SD**	**Min**	**Median**	**Max**	**CV%**	**Geometric** **Mean**
Tmax (h)	13	3.77	1.47	1.50	4.00	6.00	38.91	3.45
Cmax (ng/mL)	13	2181.38	605.49	1432.63	1944.80	3278.09	27.76	2108.30
AUC_0–8_ (ng·h/mL)	13	13,594.11	3590.73	9129.46	12,747.47	21,042.61	26.41	13,182.44
AUC_0–12_ (ng·h/mL)	13	19,050.75	4692.05	13,675.75	18,323.23	27,906.62	24.63	18,552.82
AUClast (ng·h/mL)	13	28,204.26	6115.74	21,040.18	25,706.36	39,340.37	21.68	27,628.98
AUC_0–∞_ (ng·h/mL)	13	34,529.06	7747.10	23,490.27	33,655.06	48,232.05	22.44	33,746.03
T_1/2_ (h)	13	9.01	1.86	6.42	8.91	12.11	20.65	8.83
**Day 14**								
**Variable**	***N***	**Mean**	**SD**	**Min**	**Median**	**Max**	**CV%**	**Geometric** **Mean**
Tmax (h)	12	1.79	0.66	1.00	1.50	3.00	36.60	1.69
Cmax (ng/mL)	12	5758.05	1255.01	3999.98	5408.53	7875.76	21.80	5637.93
AUC_0–8_ (ng·h/mL)	12	37,323.09	8626.89	25,221.71	34,654.74	52,790.79	23.11	36,466.47
AUC_0–12_ (ng·h/mL)	12	48,845.72	11,387.45	33,030.74	45,743.04	69,808.33	23.31	47,701.24
AUClast (ng·h/mL)	12	68,036.28	16,013.98	47,744.16	64,970.83	97,947.49	23.54	66,401.03
AUC_0–∞_ (ng·h/mL)	12	79,699.89	17,978.55	57,653.89	78,557.16	112,876.64	22.56	77915.42
T_1/2_ (h)	12	8.68	1.39	6.66	8.09	10.97	15.97	8.58

**Figure A1:**
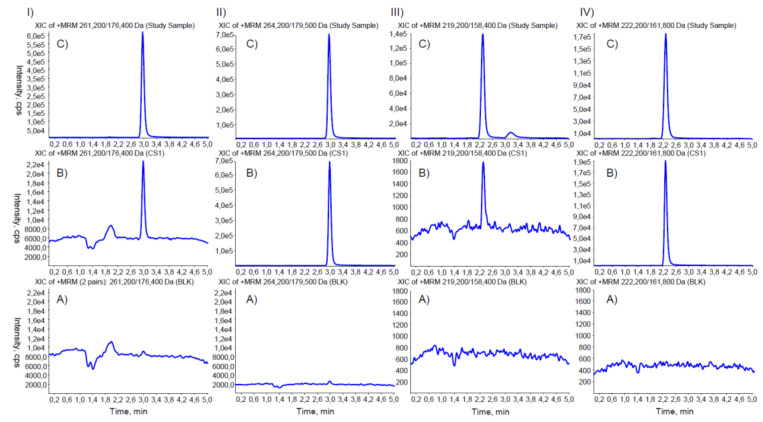
MRM chromatograms of carisoprodol (**I**) and I.S. (**II**), and meprobamate (**III**) and I.S. (**IV**), obtained from human plasma samples: (**A**) blank plasma, (**B)** blank plasma spiked at the LLOQ level, and (**C**) plasma sample from a subject after treatment with 350 mg of carisoprodol (1.0 h after dosing, determined concentration of 929.5 and 1365.1 ng/mL for carisoprodol and meprobamate, respectively).

## Data Availability

Additional details and Datasets for the data presented in this study are available from the corresponding authors upon request.

## Supplementary Materials

The following supporting information can be downloaded at: https://www.mdpi.com/article/10.3390/jcm11030858/s1, Figure S1: mean plasma concentration vs. time, carisoprodol, Day 7; Figure S2: mean plasma concentration vs. time, meprobamate, Day 7; Table S1: pharmacokinetics parameters of carisoprodol on D7; Table S2: pharmacokinetic parameters of meprobamate on D7.

## Appendix A

Concentrations of carisoprodol and meprobamate in plasma samples were determined by SFBC Anapharm Europe, Barcelona, Spain using a validated method.

Carisoprodol and meprobamate were extracted using a solid-phase extraction procedure with reversed-phase 30 mg plates and measured by reversed-phase high performance liquid chromatography coupled to a tandem mass spectrometry detector (LC/MS/MS). The detection condition of the mass spectrometer was positive ion mode with detection by multiple-reaction monitoring using the m/z transitions 261.20 to 176.40 for carisoprodol, 219.2 to 158.3 for meprobamate, and 264.2 to 179.5 and 222.20 to 161.8.10 for carisoprodol-d3 and meprobamate-d3, respectively. Quantitation was executed using the peak area ratio method. A weighted (1/X2) linear regression was performed to determine the concentration of the analytes.

Before use, the method was fully validated in compliance with the FDA Guidance for Industry (Bioanalytical Method Validation, May 2001). The linearity of standard curves was confirmed (r ≥ 0.9940) over the concentration range of 10 to 8000.00 ng/ mL for both analytes. The method showed good selectivity with no endogenous peaks present at the retention time of carisoprodol and meprobamate (Figure A1). The intra- and inter-assay accuracy, expressed as relative error, were between −13.5% and 3.24% for both analytes. The intra- and inter-assay precision were between 3.78 and 5.77% and 3.38 and 9.67 for carisoprodol and meprobamate, respectively. Other parameters like sensitivity, recovery, matrix effect, dilution integrity, carryover, reinjection reproducibility, and stability were also assessed and showed acceptable results. The validated long term stability (85 days at −20 and −80 °C) covered the periods between the blood draws and completion of the analytical determination. The study was conducted in compliance with the Principles of Good Laboratory Practice.
