# Supplementary material for: Single and Multiple Dose PK–PD Characterization for Carisoprodol. Part I: Pharmacokinetics, Metabolites, and 2C19 Phenotype Influence. Double-Blind, Placebo-Controlled Clinical Trial in Healthy Volunteers
Source: J Clin Med. 2022 Feb 6;11(3):858. doi: 10.3390/jcm11030858 (PMC8836664; doi:10.3390/jcm11030858)
Supplement: Supplementary file 1 [file jcm-11-00858-s001.zip › jcm-1557066-supplementary.pdf]

Figure S1. mean plasma concentration vs time. carisoprodol. day 7

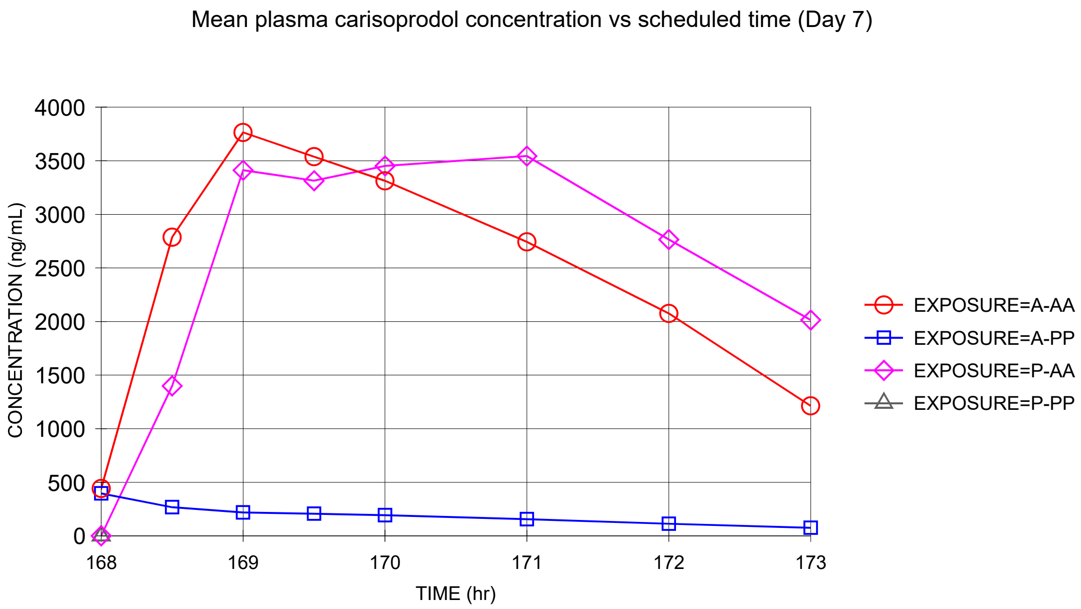

Figure S2. mean plasma concentration vs time. meprobamate. day 7

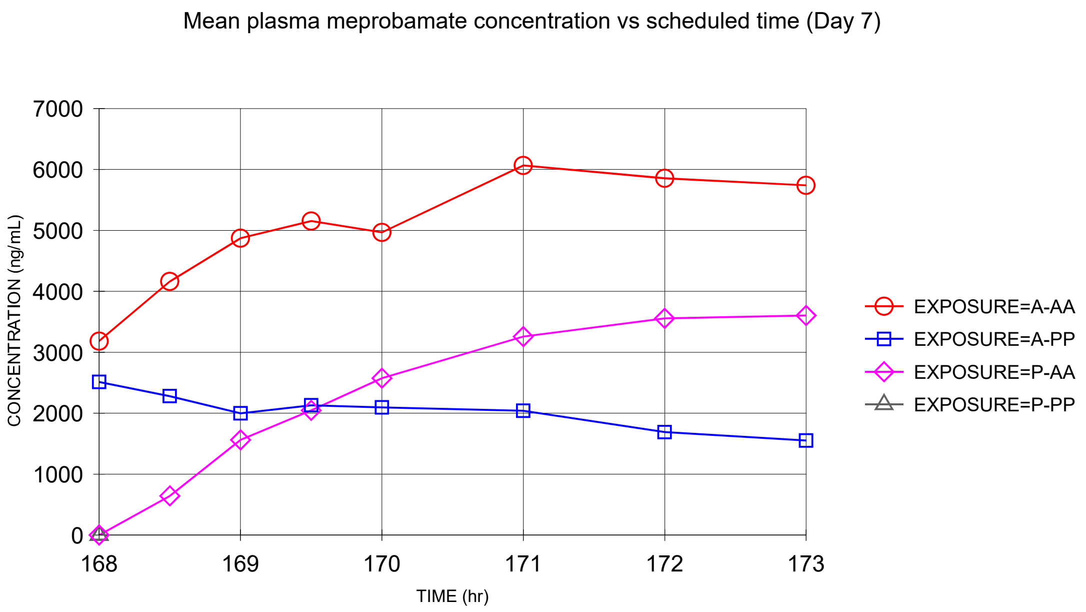

Table S1. Carisoprodol pharmacokinetic parameters at D7

CARISOPRODOL PHARMACOKINETIC PARAMETERS AFTER DOUBLE DOSING (700 MG) AT DAY 7. The group of full placebo exposure (P-PP) was not analysed									
Variable	EXPOSURE	N	Mean	SD	Min	Median	Max	CV%	Geometric _Mean
Tmax	A-AA	8	1.5625	0.7763	0.5000	1.5000	3.0000	49.6847	1.3845
Tmax	A-PP	5	0.0000	0.0000	0.0000	0.0000	0.0000	Missing	Missing
Tmax	P-AA	5	1.8000	0.8367	1.0000	2.0000	3.0000	46.4811	1.6438
Tmax	P-PP	8	N.A.	N.A.	N.A.	N.A.	N.A.	N.A.	N.A.
Cmax	A-AA	8	4790.9338	1070.5978	3170.9900	4597.7800	6369.1400	22.3463	4686.8298
Cmax	A-PP	5	396.3920	280.8761	65.6900	431.6200	817.7700	70.8582	298.0520
Cmax	P-AA	5	4167.9320	1393.3174	2939.1700	3865.2200	6495.4900	33.4295	4006.9261
Cmax	P-PP	8	N.A.	N.A.	N.A.	N.A.	N.A.	N.A.	N.A.
AUClast	A-AA	8	13064.7428	3372.4843	7717.2675	13843.8438	18500.8350	25.8136	12658.3904
AUClast	A-PP	5	895.2040	974.4737	111.2425	646.9875	2576.4350	108.8549	554.6832
AUClast	P-AA	5	13968.1490	4690.9760	9083.9750	13553.4700	20709.3625	33.5834	13356.7248
AUClast	P-PP	8	N.A.	N.A.	N.A.	N.A.	N.A.	N.A.	N.A.
AUC0-∞	A-AA	8	16915.2425	4956.6133	8340.8571	17033.8908	25317.1721	29.3026	16209.7539
AUC0-∞	A-PP	5	1228.9425	1539.9820	140.6108	702.9302	3927.4983	125.3095	686.9982
AUC0-∞	P-AA	5	21902.9243	12004.8296	11031.8209	19188.4301	40290.4198	54.8093	19489.3507
AUC0-∞	P-PP	8	N.A.	N.A.	N.A.	N.A.	N.A.	N.A.	N.A.
T1/2	A-AA	8	1.8322	0.7631	0.5913	1.9538	2.7891	41.6474	1.6561
T1/2	A-PP	5	2.0411	0.9070	1.2883	1.8015	3.5476	44.4356	1.9055
T1/2	P-AA	5	2.3591	0.7896	1.6408	2.0433	3.2157	33.4709	2.2566
T1/2	P-PP	8	N.A.	N.A.	N.A.	N.A.	N.A.	N.A.	N.A.

Table S2. Meprobamate pharmacokinetic parameters at D7.

MEPROBAMATE PHARMACOKINETIC PARAMETERS AFTER DOUBLE DOSING OF CARISOPRODOL (700 MG) AT DAY 7. The group of full placebo exposure (P-PP) was not analysed (N.A.)										
Variable	EXPOSURE	N	Mean	SD	Min	Median	Max	CV%	Geometric Mean
Tmax	P-AA	5	4.2000	0.8367	3.0000	4.0000	5.0000	19.9205	4.1289
Tmax	A-AA	8	3.4375	1.2939	1.5000	3.5000	5.0000	37.6399	3.1928
Tmax	A-PP	5	0.0000	0.0000	0.0000	0.0000	0.0000	_ _	_ _
Tmax	P-PP	8	_ _N.A.	N.A.	N.A.	N.A.	N.A.	N.A.	N.A.
Cmax	P-AA	5	3677.1240	940.8954	2325.0100	4137.2000	4560.5200	25.5878	3568.5795
Cmax	A-AA	8	6805.4400	1102.3869	5193.4300	7358.7750	7948.8800	16.1986	6722.5995
Cmax	A-PP	5	2512.4100	345.5675	1992.7800	2495.2700	2946.0400	13.7544	2492.5070
Cmax	P-PP	8	N.A.	N.A.	N.A.	N.A.	N.A.	N.A.	N.A.
AUClast	P-AA	5	12672.2300	4175.2413	6227.4400	13414.0250	17162.3400	32.9480	11991.4756
AUClast	A-AA	8	26404.2272	3822.4011	22093.5650	25720.6863	31949.5975	14.4765	26165.3379
AUClast	A-PP	5	9913.2064	1610.5459	7099.8568	10356.2800	11137.9525	16.2465	9791.5742
AUClast	P-PP	8	N.A.	N.A.	N.A.	N.A.	N.A.	N.A.	N.A.
AUC0-∞	P-AA	1	399276.3349	_ _	399276.3349	399276.3349	399276.3349	_ _	399276.3349
AUC0-∞	A-AA	4	85793.7442	33091.9454	52672.9650	79827.8169	130846.3783	38.5715	81267.7169
AUC0-∞	A-PP	5	23598.2334	9233.6900	15137.8884	20109.7479	34238.2975	39.1287	22193.3782
AUC0-∞	P-PP	8	N.A.	N.A.	N.A.	N.A.	N.A.	N.A.	N.A.
T1/2	P-AA	1	88.6257	_ _	88.6257	88.6257	88.6257	_ _	88.6257
T1/2	A-AA	4	8.6049	4.4778	5.0317	7.2675	14.8528	52.0379	7.8353
T1/2	A-PP	5	5.8361	2.6945	2.6525	5.2772	8.9250	46.1693	5.3027
T1/2	P-PP	8	N.A.	N.A.	N.A.	N.A.	N.A.	N.A.	N.A.
